# Ultraviolet Light-Assisted Copper Oxide Nanowires Hydrogen Gas Sensor

**DOI:** 10.1186/s11671-018-2566-6

**Published:** 2018-05-15

**Authors:** Nabihah Sihar, Teck Yaw Tiong, Chang Fu Dee, Poh Choon Ooi, Azrul Azlan Hamzah, Mohd Ambri Mohamed, Burhanuddin Yeop Majlis

**Affiliations:** Institute of Microengineering and Nanoelectronics (IMEN), Universiti Kebangsaan Malaysia, 43600 Bangi, Selangor Malaysia

**Keywords:** Copper oxide nanowire, Hydrogen gas sensor, UV light, Stability

## Abstract

We fabricated copper oxide nanowires (CuO NWs) ultraviolet (UV) light-assisted hydrogen gas sensor. The fabricated sensor shows promising sensor response behavior towards 100 ppm of H_2_ at room temperature and elevated temperature at 100 °C when exposed to UV light (3.0 mW/cm^2^). One hundred-cycle device stability test has been performed, and it is found that for sample elevated at 100 °C, the UV-activated sample achieved stability in the first cycle as compared to the sample without UV irradiation which needed about 10 cycles to achieve stability at the initial stage, whereas the sample tested at room temperature was able to stabilize with the aid of UV irradiation. This indicates that with the aid of UV light, after some “warming up” time, it is possible for the conventional CuO NW sensor which normally work at elevated temperature to function at room temperature because UV source is speculated to play a dominant role to increase the interaction of the surface of CuO NWs and hydrogen gas molecules absorbed after the light exposure.

## Background

In the recent developments, various types of gases such as hydrogen, ammonia, butane, and carbon monoxide have been extensively used in industries [1–6].Various metal oxide such as copper oxide, tin oxide, and zinc oxide-based gas sensors have been studied extensively due to their advantages, for instance, low fabrication cost and high sensitivity towards hazardous gases [7, 8]. Copper oxide nanowire (CuO NW) is an emerging material which is suitable for gas sensor due to its high surface to volume ratio to enhance the performance of the sensor [9, 10]. There have been reported that CuO NWs are suitable for detection of various gases such as ethanol, butane, carbon monoxide, ammonia, and nitrogen oxide [9–11]. Generally, metal oxide-based gas sensors required high operating temperature to achieve excellent sensing performance. However, high operating temperature could lead to high power consumption. Also, prolong operation at high temperatures has drift problems caused by sintering and diffusion process [12, 13]. Therefore, exploring a new method without enhancement of metal oxide-based gas sensors has been explored to minimize this problem.

Surface functionalization by decorating the material surfaces with nanoparticles to enhance the sensitivity and selectivity of the gas sensor towards the hazardous gas using chemical route has been reported elsewhere [14, 15]. However, this method is very complicated as the preparation involved many chemicals and consuming lots of reaction time to tune the desired size of the nanoparticles for the aimed applications [16]. To avoid this complication, some researchers are attempting to enhance the performance of gas sensor by using UV light to create the photogenerated electron and hole pairs as an alternate route because it does not require complicated chemical preparation and hence time efficiency [17–20]. Moreover, the use of UV light could be the alternate solution to avoid heating the sensor and results in reducing the power consumption and avoiding the sensor degradation due to the high temperatures as conventional gas sensor required high temperature to operate and achieve stability.

Report on the use of UV-enhanced indium oxide and tin dioxide sensors have been studied by Comini et al. [20]. It has been reported that the UV light enhanced the sensing performance of the CO and NO_2_ gas sensor at room temperature which increases the reaction of the interaction on the semiconductor. Besides that, Comini et al. also reported that UV light can reduce the poisoning effect of gas sensing which changes the electrical properties quite irreversible by enhancing the desorption process [20]. In addition, the exposure of UV irradiation has been reported to improve the sensitivity, stability, and response time of ethanol gas sensor by Gong et al. [21]. Moreover, the gas sensor also could operate at relatively lower temperature that might be attributed to the conducting electrons in the ZnO nanowires generated by UV irradiation that promote the reduction of ethanol and hence lead to higher sensitivity and less response time. Therefore, irradiation of UV light is one of the suitable methods to enhance the performance of the gas sensor.

To our knowledge, the effect of UV light toward the hydrogen gas (H_2_) sensing properties of CuO NWs has not been reported yet. Therefore, our study focused on the effect of activated UV light toward the hydrogen gas-sensing properties of CuO NWs at room temperature and 100 °C. The CuO NWs are grown by using thermal oxidation method because this technique exhibits higher crystallinity and longer aspect ratios compared to the other reported methods such as solution-based routes.

## Results and Discussions

**Fig. 1:**
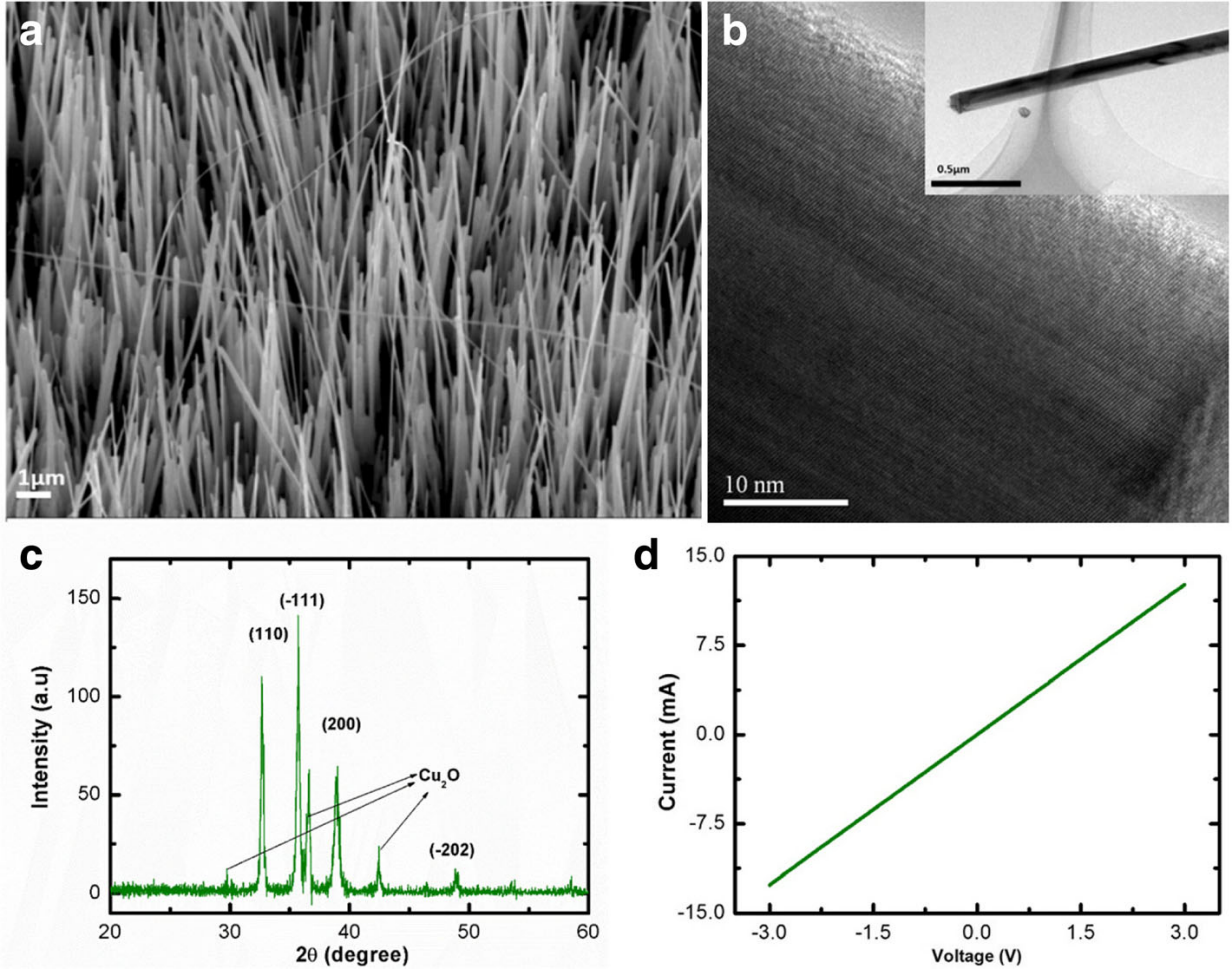
**a** FESEM image of the grown CuO NWs. **b** TEM image of CuO. **c** X-ray diffraction profile of CuO nanowires. **d** IV characteristics of CuO and Pt

The morphology of grown CuO NWs is shown in Fig. 1a. The image shows that most of the NWs are quite aligned and perpendicular to the surface of the substrate. Meanwhile, Fig. 1b shows the TEM image of highly crystalline CuO NWs obtained. The inset images show that the diameter of the CuO NW is 120 nm. XRD pattern in Fig. 1c matches the database card: JCP2:00-045-0937 for CuO and JCP2:00-005-0667 for Cu_2_O. It shows that the synthesized CuO NWs have monoclinic structure. Figure 1d shows I-V characteristic of CuO NWs on Pt electrodes with Ohmic behavior since Pt has higher work function (6.35 eV) compared to CuO (5.2 eV). This agrees with the fact of Ohmic behavior is obtained when p-type semiconductor contact with higher work function material [22]. Table 1 shows the sensor response and stability of hydrogen gas sensor with and without the presence of UV at two different temperatures (room temperature and 100 °C). The sensor response is defined as *S* = $\frac{I_g-{I}_a}{I_a}\times 100\%$, where *I*_*g*_ is the current flow after the sample is exposed to the gas and *I*_*a*_ is the initial current before exposed to the gas [23, 24].

**Table 1:** Sensor response and stability of hydrogen gas sensor

Temperature (^°^C)	Presence of UV	Sensor response, *S* (%)	Stability
Room temperature	No	0.0041	Bad
Room temperature	Yes	0.0527	Quite good
100 °C	No	4.2844	Very good
100 °C	Yes	4.6012	Very good

**Fig. 2:**
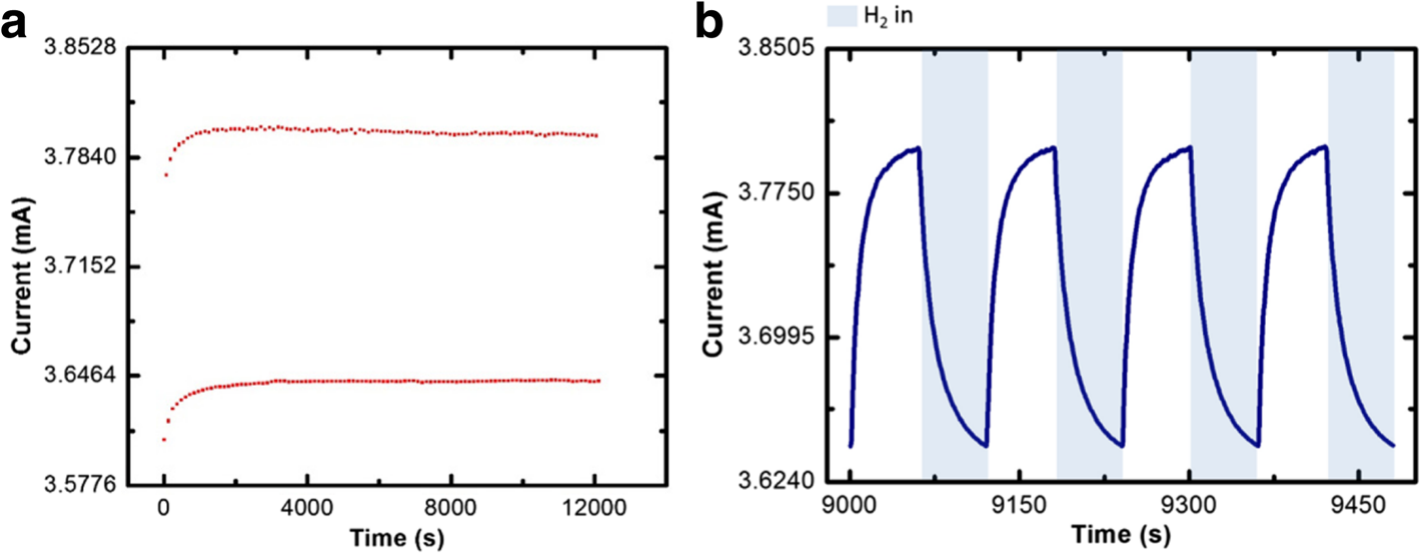
Hydrogen gas sensing behaviour in the dark at 100 ^°^C. **a** Maximum and minimum point for 100 cycles. **b** Response of CuO NWs sensors toward hydrogen gas

**Fig. 3:**
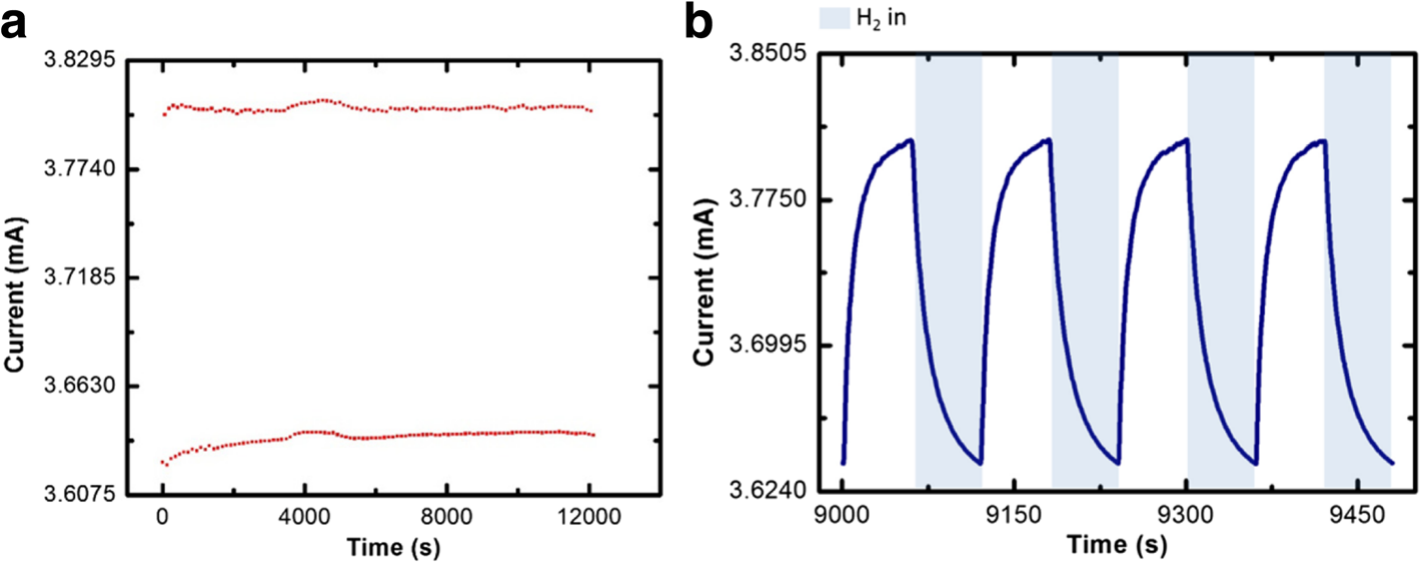
Hydrogen gas sensing behaviour for UV activated sensor working at 100 ^°^C. **a** Maximum and minimum point for 100 cycles. **b** Response of CuO NWs sensors toward hydrogen gas

Figures 2 and 3 show the gas response in the dark and under UV illumination at 100 °C respectively. The results show that the responses of the gas sensors are stable. During operating temperature at 100 °C, electron concentration for the sensing reaction increases due to the sufficient thermal energy at higher temperature to overcome the potential barrier [25]. Introducing the 365-nm UV light has significantly improved the sensing stability of CuO NW sensor at the initial stage of sensing cycle. The UV-activated sample could achieve stability in the first cycle, compared to the sample without UV irradiation which needed about 10 cycles to achieve stability at the initial stage. Furthermore, sensor response of the gas sensor increases with the UV light irradiation (~ 4.6%) compared to without UV light irradiation (~ 4.3%) as shown in Table 1. Therefore, the results show that low power LED could increase the sensor response with the same temperature without an increase in the heating temperature which can lead more power loss.

**Fig. 4:**
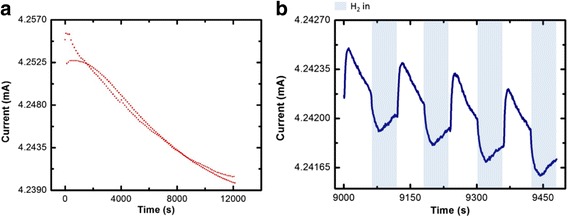
Hydrogen gas sensing behaviour in the dark at room temperature. **a** Maximum and minimum point for 100 cycles. **b** Response of CuO NWs sensors toward hydrogen gas

**Fig. 5:**
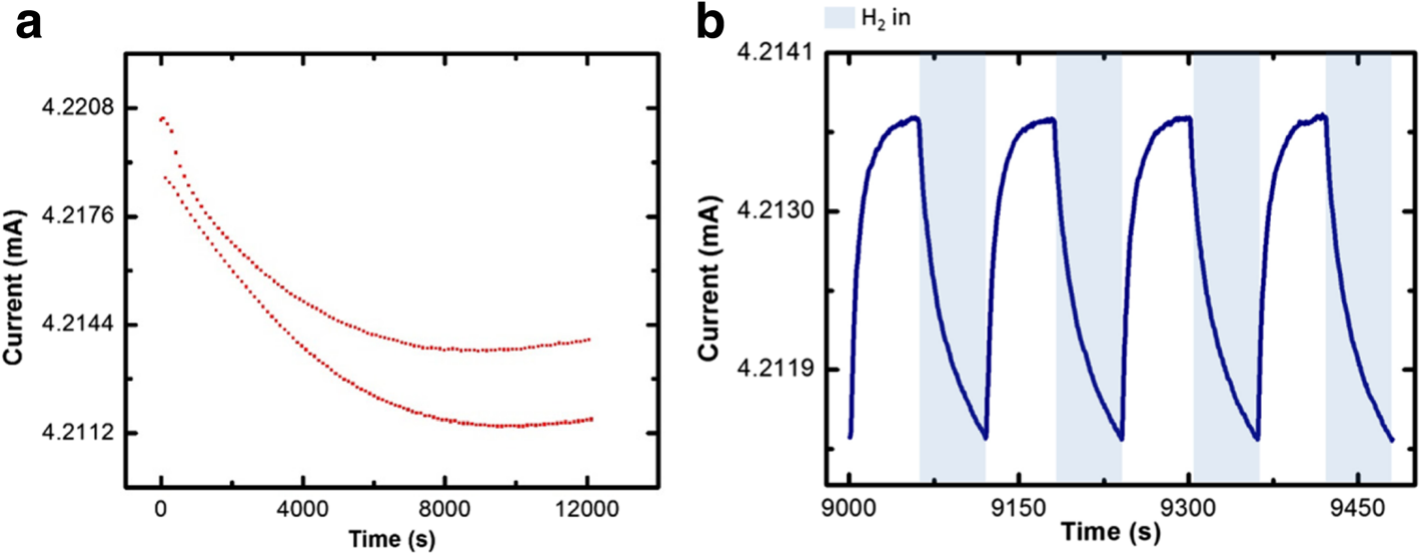
Hydrogen gas sensing behaviour for UV activated sensor at room temperature. **a** Maximum and minimum point for 100 cycles. **b** Response of CuO NWs sensors toward hydrogen gas

Figures 4 and 5 present the gas response in the dark and under UV illumination at room temperature. Figure 5 shows that the stability of gas sensor is quite good at room temperature with the help of UV light after some time. The sensor starts to stabilize at about 9000 s. Figure 5b shows an enlarged graph from 9000 to 9500 s. The increase in sensor response is observed from 0.0041 to 0.0527% without and with assist of UV light. Compared to Fig. 4 without the UV irradiation at room temperature, there is no sign of stabilization until the end of test (12,000 s). The sensor with exposure of UV light is required about 2.5 h to “warming up” and then it can work in stable condition after that. Compared to the sensor without the assist of UV, the conductivity keeps reducing and the response is unstable. This might be due to the memory effect and sensor poisoning which normally occur at room temperature [26, 27]. The result shows that the sensor has a potential to operate at a room temperature with the assist of UV without the use of power hungry heating element.

The sensing mechanism of hydrogen gas sensor without the irradiation of UV light is explained below. Hydrogen gas is interacting with CuO NWs through the pre-adsorbed oxygen ions on the CuO NWs surfaces when the CuO NWs is exposed to hydrogen as described in Eqs. (1) and (2). The free electrons release to CuO NWs due to the reactions between H_2_ molecules and pre-adsorbed oxygen ions and recombine with the holes in CuO NWs [Eq. (3)]. This process is resulting the decrease of hole concentration in the nanowires and increase in the resistance [28].

1 $$ {\mathrm{H}}_2\left(\mathrm{ads}\right)+{\mathrm{O}}^{-}\ \left(\mathrm{ads}\right)\leftrightarrow {\mathrm{H}}_2\mathrm{O}+{\mathrm{e}}^{-} $$

2 $$ {\mathrm{H}}_2\left(\mathrm{ads}\right)+{\mathrm{O}}^{2-}\left(\mathrm{ads}\right)\leftrightarrow {\mathrm{H}}_2\mathrm{O}+{2\mathrm{e}}^{-} $$

3 $$ {\mathrm{e}}^{-}+\mathrm{h}\bullet \leftrightarrow \mathrm{Null} $$

Upon irradiation of UV light, the photogenerated electron and hole pairs are created. When the gas sensor is exposed to UV light, photogenerated holes contribute into the conduction process; meanwhile, photogenerated electrons migrate to the surface [29]. Therefore, more electrons are available for the chemisorption of O_2_. Furthermore, UV light affects the chemisorption and desorption processes by altering the energetic state of H_2_ gas on the CuO NW surface [30, 31]. The whole process speeds up the interaction between the surface of the CuO NWs and H_2_ gas and increases the reaction.

## Conclusion

As a conclusion, the CuO NWs have been successfully grown by using thermal oxidation method which is cheap, simple, and non-catalyst method. Hydrogen gas sensor with the assistance of UV light has been successfully fabricated. The dominant role of UV light is to enhance the gas sensing properties for room temperature and 100 °C. The stability of the gas sensor is very good at 100 °C where it is in elevated operating temperature. With the irradiation of UV light, sensor could achieve stability in the first cycle compared to sensor without UV light which required about 10 cycles to achieve stability at initial stage. The sensor response also increases with the assistance of UV light. Therefore, we are not required to use higher operating temperature which consumes high power. Besides that, we have found that the sensor with irradiation of UV light has a great potential to operate at low temperature (room temperature) which can reduce the loss power consumption. The stability and sensor response increase with the assistance of UV light.

## Method/Experimental

Cr/Pt electrodes chip is fabricated through photolithography process. The thermal oxide (150 nm) layer was grown on silicon substrate as an insulating layer under 1500 sccm oxygen flow at 1000 °C for 3 h. Then, the substrate was spin-coated with 1 μm thick of positive photoresist (Futurrex PR1-1000A) at 3000 rpm for 40 s. Soft-baked process was taken place before UV exposure at 120 °C for 120 s. Next, chrome photomask with interdigitated electrode pattern was transferred to the sample with exposure of ultraviolet light (356 nm) under the intensity of 2.41 mW/cm^2^ with 50 s exposure time. Cr/Pt (10 nm/150 nm) contact electrodes were deposited on the sample using magnetron sputtering system NanoFilm and sputter coater Baltec SCD 005 at 2.0 × 10^− 5^ Torr respectively. Lift-off process had been conducted to attain the expected electrodes pattern.

**Fig. 6:**
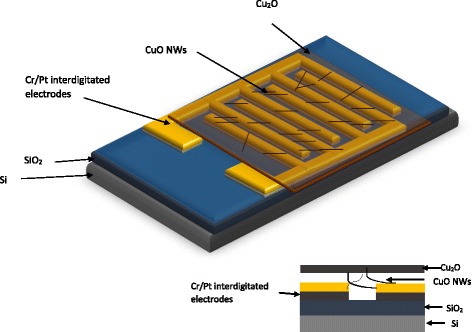
3D device structure of gas sensor

In this experiment, CuO NWs were grown on copper foil by using a thermal oxidation process. This growth process takes 6 h with 1500 sccm gas flow at 600 °C in oxygen ambient. The grown CuO NWs were characterized by field emission scanning electron microscope (FESEM) Carl Zeiss Supra 55 VP, transmission electron microscope (TEM) JOEL JEM-2010, and X-ray diffraction (XRD) Bruker D8 Advance. Then, the gas sensor is fabricated using the thermally grown CuO NWs. The layer of CuO NWs and Cu_2_O thin film was transferred on top of platinum electrodes with the layer of Cu_2_O facing on top and CuO NWs facing IDT electrodes as depicted by inset in Fig. 6. The active layer was formed by the Cu_2_O and CuO NWs; nevertheless, the sensing response is mainly due to CuO NWs rather than Cu_2_O thin film because of the higher surface to volume ratio aspect of nanowires. Here, the layer of Cu_2_O was formed indirectly during the high-temperature oxidation and annealing process. After that, annealing process was taken place at 400 °C for 20 min in N_2_ ambient to enhance the contact between the CuO NWs and interdigitated electrodes. The overall device structure design is illustrated in Fig. 6.

Next, the sensor was placed in the chamber during the sensing process followed by the mass flow controller that controlled the flow of the 100 ppm hydrogen (H_2_) gas and purified air purchased from Air Products into the chamber for 100 cycles at room temperature and 100 °C with and without the exposure of 3 mW/cm^2^ intensity UV. The UV source was generated by the 365 nm wavelength, 3.8 V and 2 mW LED purchased from VioLed International Inc. with serial number 370-5C90. The current response was monitored by two probe measurements placed on the sensor device with the data acquisition system. The current-voltage (I-V) characteristics were then measured by sweeping the applied voltage from − 3 to 3 V in dark ambient at room temperature by using Keithley 2400 power source. The same sensing response measurement was performed at 100 °C on top of the heating stage (ATV Technologie Gmbh, TR-120 D). A complete sensing cycle will be conducted in the sequence of air-H_2_-air at a constant flow rate of 50 sccm.
